# Chemical Component and Proteomic Study of the *Amphibalanus* (= *Balanus*) *amphitrite* Shell

**DOI:** 10.1371/journal.pone.0133866

**Published:** 2015-07-29

**Authors:** Gen Zhang, Li-sheng He, Yue-Him Wong, Ying Xu, Yu Zhang, Pei-yuan Qian

**Affiliations:** 1 Environmental Science Programs and Division of Life Science, School of Science, The Hong Kong University of Science and Technology, Clearwater Bay, Kowloon, Hong Kong SAR, R. P. China; 2 Sanya Institute of Deep-Sea Science and Engineering, Chinese Academy of Science, Sanya City, Hainan Province, 572000, P. R. China; 3 Shenzhen Key Laboratory of Marine Bioresource and Eco-Environmental Science, College of Life Science, Shenzhen University, Shenzhen, 518060, P. R. China; CSIR- National institute of oceanography, INDIA

## Abstract

As typical biofoulers, barnacles possess hard shells and cause serious biofouling problems. In this study, we analyzed the protein component of the barnacle *Amphibalanus* (= *Balanus*) *amphitrite* shell using gel-based proteomics. The results revealed 52 proteins in the *A*. *Amphitrite* shell. Among them, 40 proteins were categorized into 11 functional groups based on KOG database, and the remaining 12 proteins were unknown. Besides the known proteins in barnacle shell (SIPC, carbonic anhydrase and acidic acid matrix protein), we also identified chorion peroxidase, C-type lectin-like domains, serine proteases and proteinase inhibitor proteins in the *A*. *Amphitrite* shell. The sequences of these proteins were characterized and their potential functions were discussed. Histology and DAPI staining revealed living cells in the shell, which might secrete the shell proteins identified in this study.

## Introduction

Biomineralization is a widespread phenomenon in organisms. The variety of solid inorganic structures formed by biomineralization are used for support, protection, mastication, gravity perception, and various other functions [1].

The barnacle, which is a crustacean, has a hard shell that is a result of biomineralization. Unlike other crustaceans (for instance, shrimp and crab), for which the shells are periodically shed and rebuilt for the purposes of growth, regeneration, metamorphosis, and reproduction [2, 3], barnacle shells grow continously throughout their life, and only the interior cuticle is moltted to make more space for the softbody. The barnacle shell consists of a chitin-protein microfibril framework and is mineralized with calcite [4]. The inner lamina of barnacle shell consists of parallel calcified layers separated by organic sheets, and these sheets show autofluorescence and consist of chitin surrounded by proteoglycans and other minor proteins [5].

So far, only a few studies have focused on the protein component of barnacle shells. Khalifa et al. [4] revealed highly acidic proteins in the matrix components of the *Amphibalanus* (= *Balanus*) *amphitrite* shell. A glycoprotein, namely settlement inducing protein complex (SIPC) is also present in barnacle shell [6–8]. Khandeparker and Anil [9] charaterized arthropodin protein complex from barnacel extracts and identified two undescribed subunits (66-kDa and 98-kDa) in barnacle shell. In the basal plate of *Magabalanus rosa*, Kamino's group solubilized the proteins of secondary cement and found that the cement is composed of at least two distinct groups of proteins (formic-acid-insoluble proteins and highly hydoxylated proteins) [10]. Later, they further identifed several cememts proteins (e.g. Mrcp-68k, Mrcp-100k, Mrcp-52k [11] and Mrcp-19k [12]) from the basal plate.

In the present study, we sought to profile the chemical and protein components of the adult shell of the typical intertidal barnacle *A*. *amphitrite* using X-ray fluorescence (XRF) analysis and gel-based proteomics. An understanding of the proteome and clarification of its roles in the barnacle shell will contribute more information to practical engineering processes and the synthesis of novel materials [13].

## Results

### X-ray fluorescence analysis of the barnacle shell

The XRF analysis revealed calcium as the major component of the *A*. *amphitrite* shell, occupying more than 92% of both the weight and molar percentages. Small amounts of Na, Mg and Sr were also detected in the shell (Table 1 and S1 Fig).

**Table 1:** Results for the *Amphibalanus amphitrite* shell using the XRF-technique (quantitative analysis).

Element	Line	Energy	ms%	mol%	K	Net	Error%
Na	K	1.04	1.3994	2.4013	0.0030933	870	13.1503
Mg	K	1.25	0.9925	1.6104	0.0035363	2016	2.0118
S	K	2.31	1.3228	1.6275	0.0185220	51170	0.0549
Cl	K	2.62	1.3704	1.5249	0.0184698	41130	0.1255
Ca	K	3.69	93.8160	92.3411	0.7592769	1638546	0.1926
Sr	K	14.14	1.0989	0.4948	0.0184209	63678	0.1099

### Proteomics analysis of the barnacle shell

**Fig 1:**
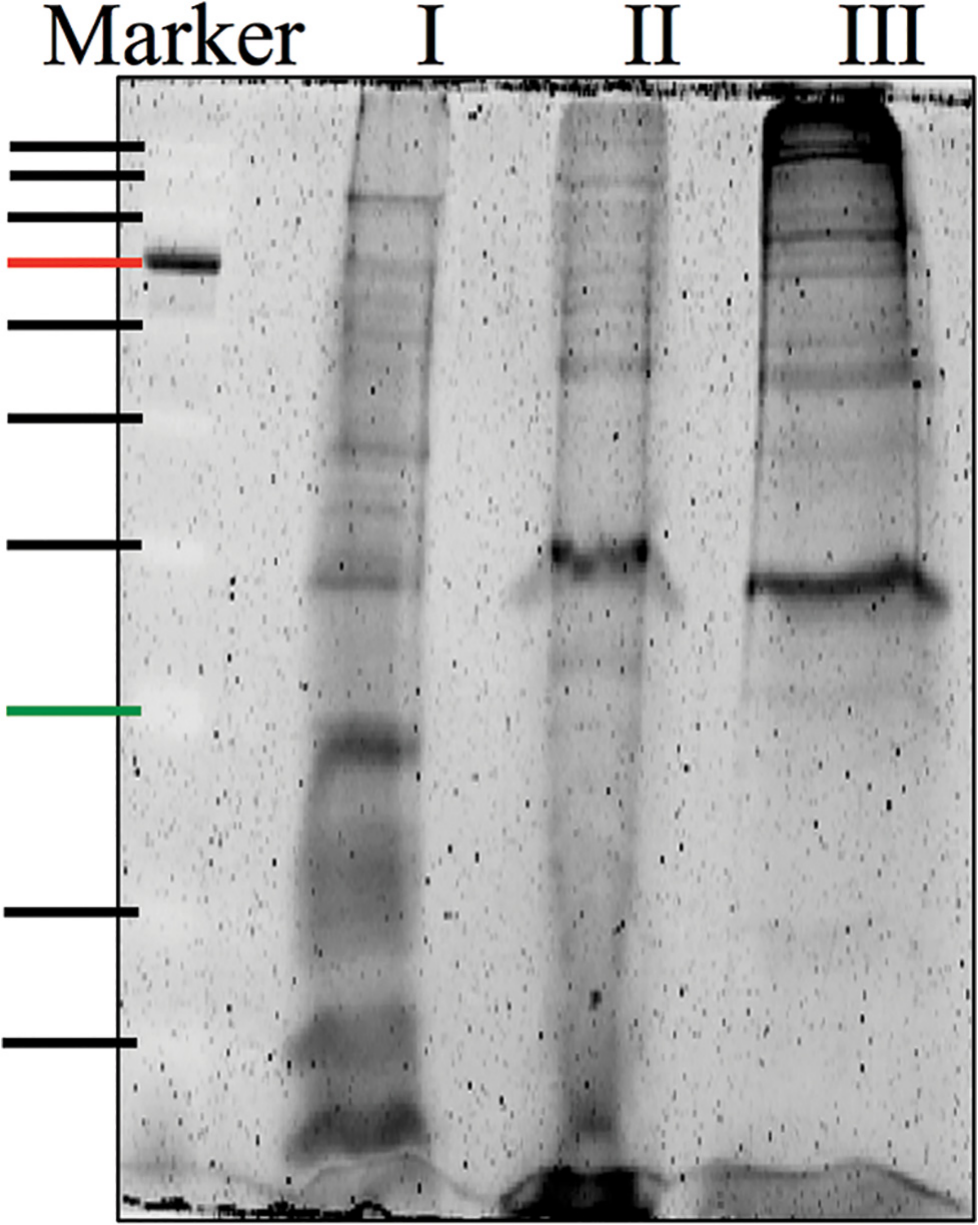
PAGE image showing protein extracts of the *Amphibalanus amphitrite* shell. The gel was stained by SYPRO Ruby dye (Invitrogen) and the later coomassie blue staining confirmed the molecular weight of each band in the marker. Lane I: acetic acid fraction; Lane II: 1% SDS fraction; Lane III: 10% SDS fraction. Marker from top to bottom: 170, 130, 93, 70, 53, 41, 30, 22, 14 and 9 kDa.

The total protein from whole barnacle shell was extracted in acetic acid, 1% SDS buffer, and 10% SDS buffer sequentially. Three fractions were collected and analyzed independently. The PAGE gel revealed clear bands for the extracts from the barnacle shell (Fig 1). The combination of MS results from all three fractions led to the identification of a total of 52 proteins in our transcriptome database (S2 Fig). Among these proteins, 20, 3, and 12 were uniquely present in the acetic acid, 1% SDS, and 10% SDS fractions, respectively. Six proteins were shared by all three fractions. Based on the KOG database, 40 proteins were categorized into 11 functional groups; the remaining 12 proteins were considered as hypothetical proteins (S1 Table).

### Settlement-inducing protein complex

The settlement-inducing protein complex (SIPC) is a glycoprotein and has been previously detected in the *A*. *amphitrite* shell by Western blot analysis [6]. MS analysis detected SIPC in both the 1% and 10% SDS fractions but not in the acetic acid fraction.

### Shell matrix proteins

**Fig 2:**
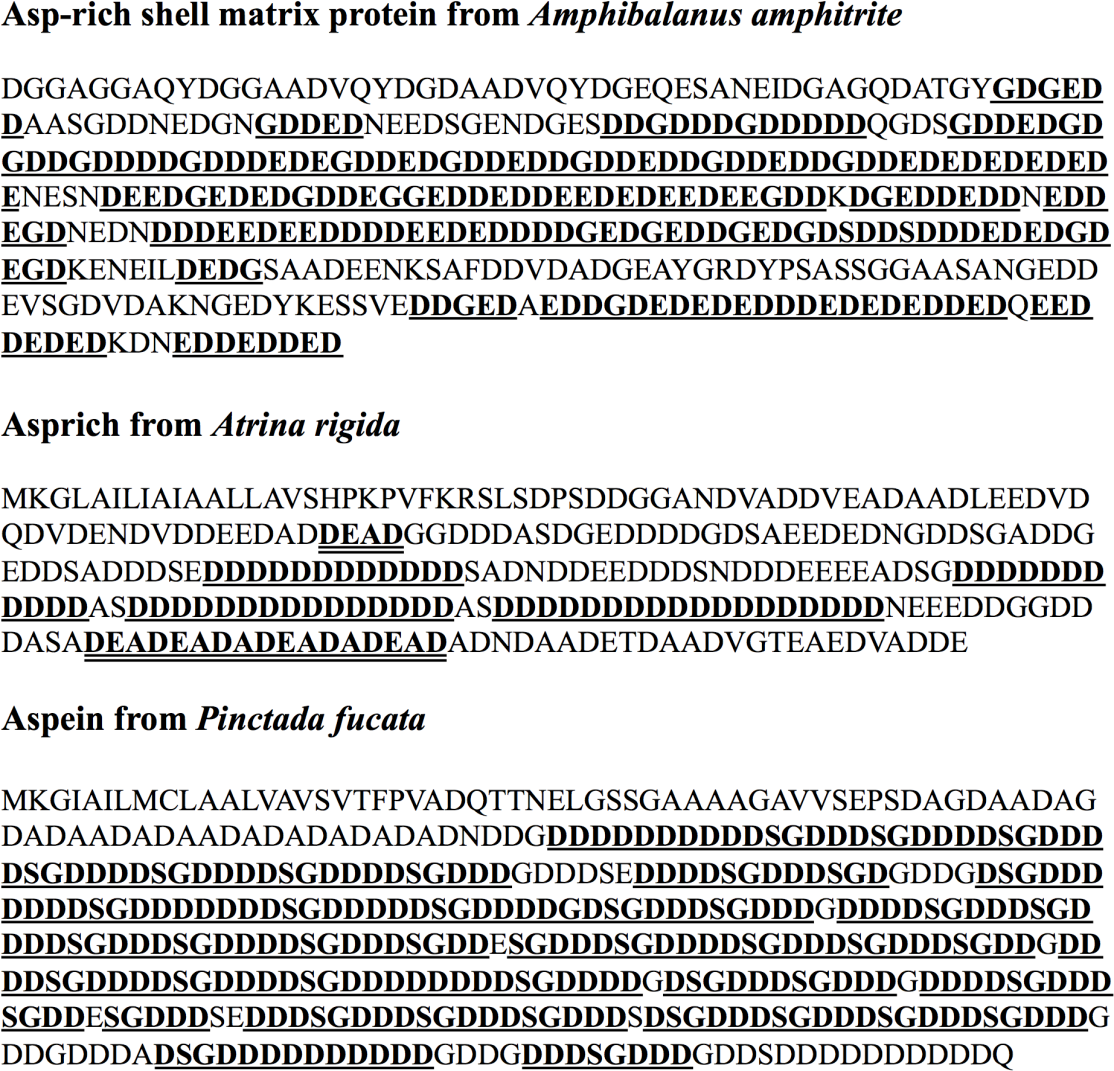
Comparison of an Asp-rich shell matrix protein from *Amphibalanus amphitrite* with Asprich (*Atrina rigida*; AAU04808.1) and Aspein (*Pinctada fucata*; AB094512). The Asp-rich shell matrix protein from *A*. *amphitrite* mainly contained Asp punctuated with Glu or Gly, which are labeled in bold with a single underline. Asprich includes three poly(Asp) motifs (presented in block with a single underline) and two DEAD motifs (presented in bold with a double underline). Aspein consists of mostly poly(Asp) blocks punctuated with Ser-Gly dipeptides (presented in bold with a single underline).

An acidic shell matrix protein (coded by CL6615.Contig1_Ba_mix) was identified in the acetic acid fraction (Fig 2). This protein did not retrieve any known proteins using Blastp or tBlastn in NCBI. The partial sequence (381 amino acids) was used to calculate the most dominant amino acids, where were Asp (41.2%), Glu (21.8%) and Gly (14.2%) (S2 Table). The pI was estimated at 2.68. Compared with Asprich from *A*. *rigida*, this protein displayed an identity of 38.6% and a similarity of 49.0%; a slightly lower similarity (31.8%) and identity (43.7%) were obtained for this protein compared to Aspein from *P*. *fucata*.

Another protein coded by the contig CL4062.Contig1_Ba_mix was detected in all three fractions. This protein was annotated as similar to a known matrix protein called Prisilkin-39 with an identity of 44%. Based on its partial sequence, this protein had a high ratio of Gly (19.4%), Tyr (20.8%) and Ser (11.8%) in sequence component. Similarly to oyster Prisilkin-39 (ACJ06766.1, pI = 8.83), this protein has a basic isoelectric point (pI = 9.75).

### Serine proteases and serine protease inhibitor

**Fig 3:**
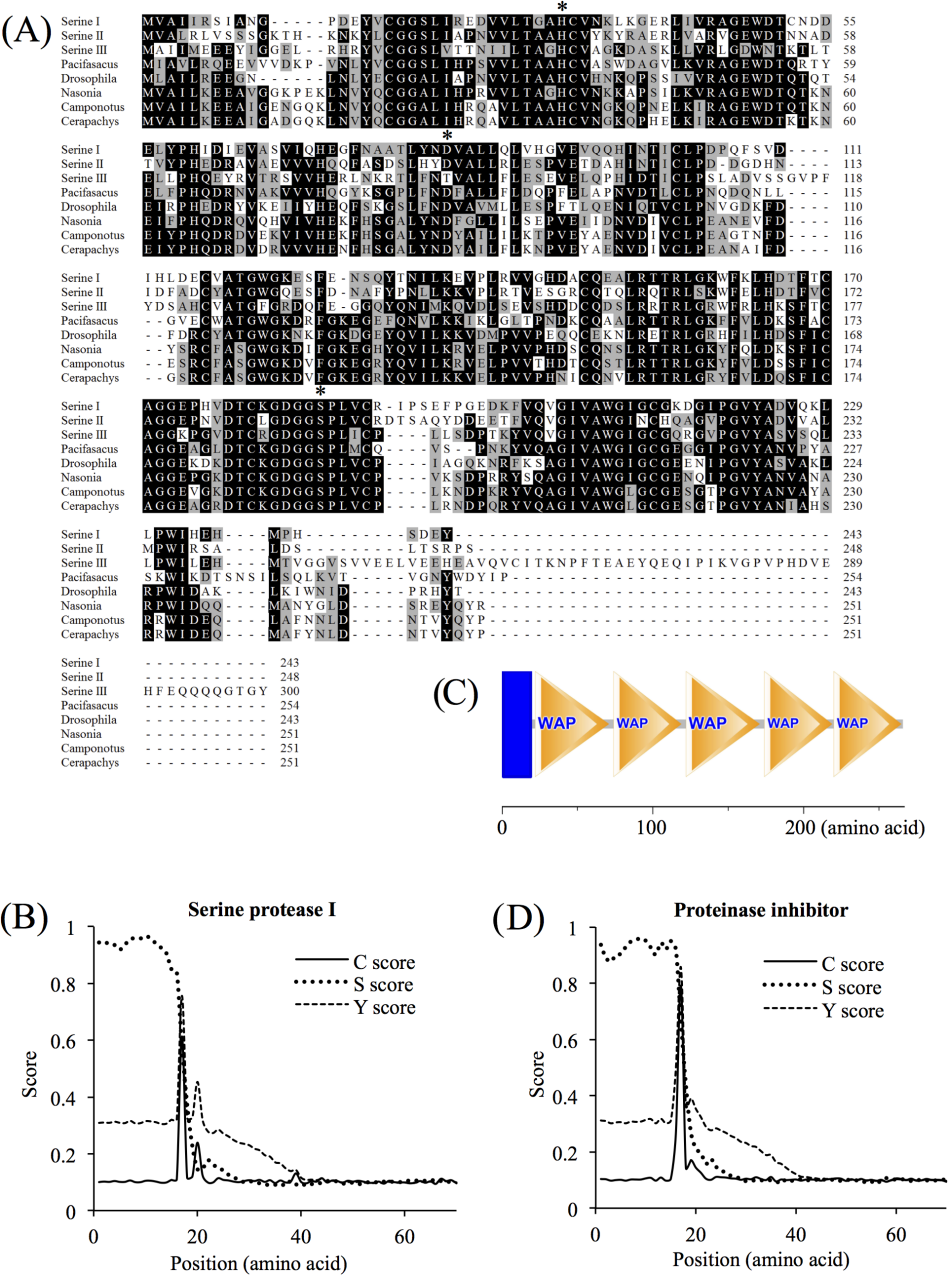
Sequence analysis of serine proteases and chorionic proteinase inhibitor from *Amphibalanus amphitrite*. (A). Alignment of three serine proteases from *A. amphitrite* (I, II, and III) with *Pacifastacus leniusculus* (ACB41380), *Drosophila sechellia* (XP002036473.1), *Nasonia vitripennis* (NP001155060), *Camponotus floridanus* (EFN72618.1), and *Cerapachys biroi* (EZA53191.1). The three catalytic triad active-site residues (H, D and S) were all conserved in the three serine protease isoforms from *A. amphitrite* and marked with an asterisk. (B). Signal peptide prediction in serine protease I from *A. amphitrite*. The first 16 amino acids were predicted to be a signal peptide. C-score (raw cleavage site score), S-score (signal peptide score) and Y-score (combined cleavage site score) were calculated using the SingalP 4.1 Server. (C). Motif scanning revealed that the chorionic proteinase inhibitor from *A. amphitrite* contained 1 transmembrane domain (blue rectangle) and 5 WAP (whey acidic protein) domains (yellow triangle). (D). Signal peptide prediction suggested that the first 16 residues represented a signal peptide in the chorionic proteinase inhibitor.

In the 1% and 10% SDS fractions, 3 different proteins were revealed to contain a trypsin-like serine protease domain by motif scanning. These proteins were named serine protease I, II, and III (Table 2). Among them, serine protease I and II were much longer than serine protease III. After alignment, the serine protease domains of these three proteins revealed a high similarity to the serine protease from *Pacifasacus*, *Drosophila*, *Nasonia*, *Camponotus* and *Cerapachys* (Fig 3A). Three catalytic triad active-site residues (H, D and S) [14] were all conserved in these three serine protease isoforms (Fig 3A). Prediction of the signal peptide revealed that the first 16 amino acids (MMRWVLLASLAALASS) were a signal peptide of serine protease I. However, no signal peptide was detected in serine protease II or III (Fig 3B).

**Table 2:** Physical and chemical parameters of three serine protease isoforms and chorionic proteinase inhibitor in *Amphibalanus amphitrite*.

	Serine Protease I	Serine protease II	Serine protease III	chorionic proteinase inhibitor
Contig	CL563.Contig1	CL1641.Contig1	CL200.Contig3	CL5577.Contig1
Present fractions	10% SDS	10% SDS	1% SDS	acetic acid
Sequence length (AA)	659	890	300	274
MW (kDa)	66.0	91.5	33.2	29.6
pI	4.88	5.74	5.44	7.29
Instability index	30.38 (stable)	34.28 (stable)	44.00 (unstable)	40.25 (unstable)
Signal peptide	yes	no	no	yes

In the acetic acid fraction, the protein coded by the contig CL5577.Contig1_Ba_mix displayed 29% similarity with the chorionic proteinase inhibitor from *Tribolodon hakonensis*. Although the identification score of this protein was only 35, MS^2^ detected 11 peptides matching with this protein. Based on the sequence in our transcriptome database, 5 continuous whey acidic protein (WAP) domains were present in the corresponding protein (Fig 3C). The WAP domain possesses peptidase inhibitor activity [15], and therefore, this contig was considered to be a protease inhibitor in the barnacle shell. The first 16 amino acids (MASWIVLLLLAPAISA) were predicted as a signal peptide using the SignalP 4.1 Server (Fig 3D).

### Shell hardening-related proteins

**Fig 4:**
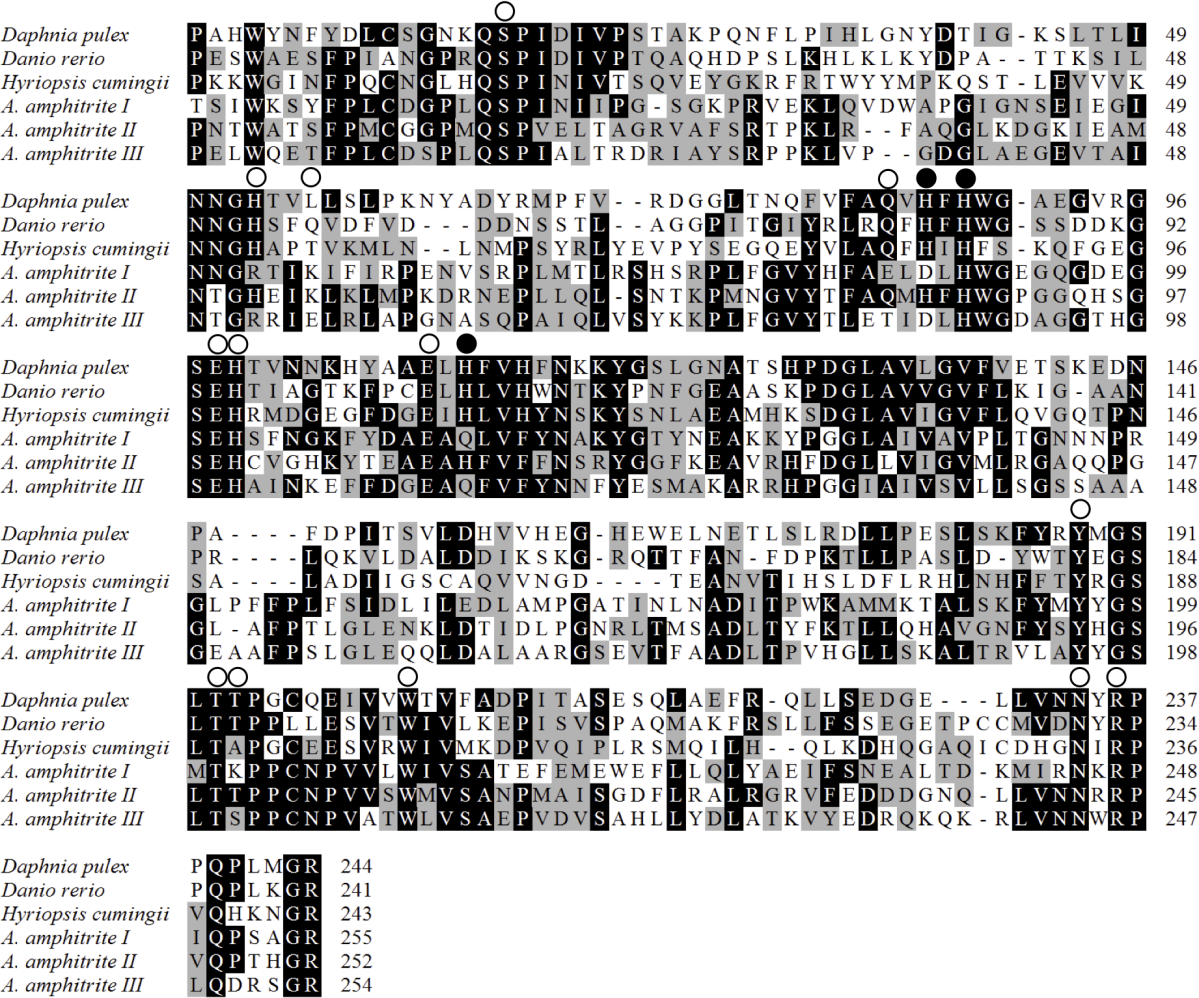
Alignment of the carbonic anhydrase from *Amphibalanus amphitrite* (I, II and III), *Daphnia pulex* (EFX81683.1), *Danio rerio* (NP571185.1) and *Hyriopsis cumingii* (AHY35316.1). Putative histidine residues that function as zinc ligands are marked with filled circles above the residues. The residues forming the hydrogen-bonded network to zinc-bound solvent molecules are depicted by open circles.

In the barnacle shell, three homologs of carbonic anhydrase were detected. The first one (coded by CL15286.Contig1_Ba_mix; *A*. *amphitrite* I) was detected in all three fractions. Its partial sequence, containing 278 amino acid residues, showed 30% identity with alpha-carbonic anhydrase from *Daphnia pulex* (EFX81683.1). The second (coded by CL10121.Contig1_Ba_mix; *A*. *amphitrite* II) and third (coded by Unigene27659_Ba_mix; *A*. *amphitrite* III) proteins both showed 26% and 30% identity with *D*. *pulex* alpha-carbonic anhydrase, respectively, and were found in the 1% and 10% SDS fractions. All three putative histidine residues that function as zinc ligands [16] were conserved in carbonic anhydrase II, similarly to the majority of the residues forming the hydrogen-bond network to zinc-bound solvent molecules (Fig 4). However, for carbonic anhydrase I and III, two of the three histidines that function as zinc ligands were mutated (Fig 4).

**Fig 5:**
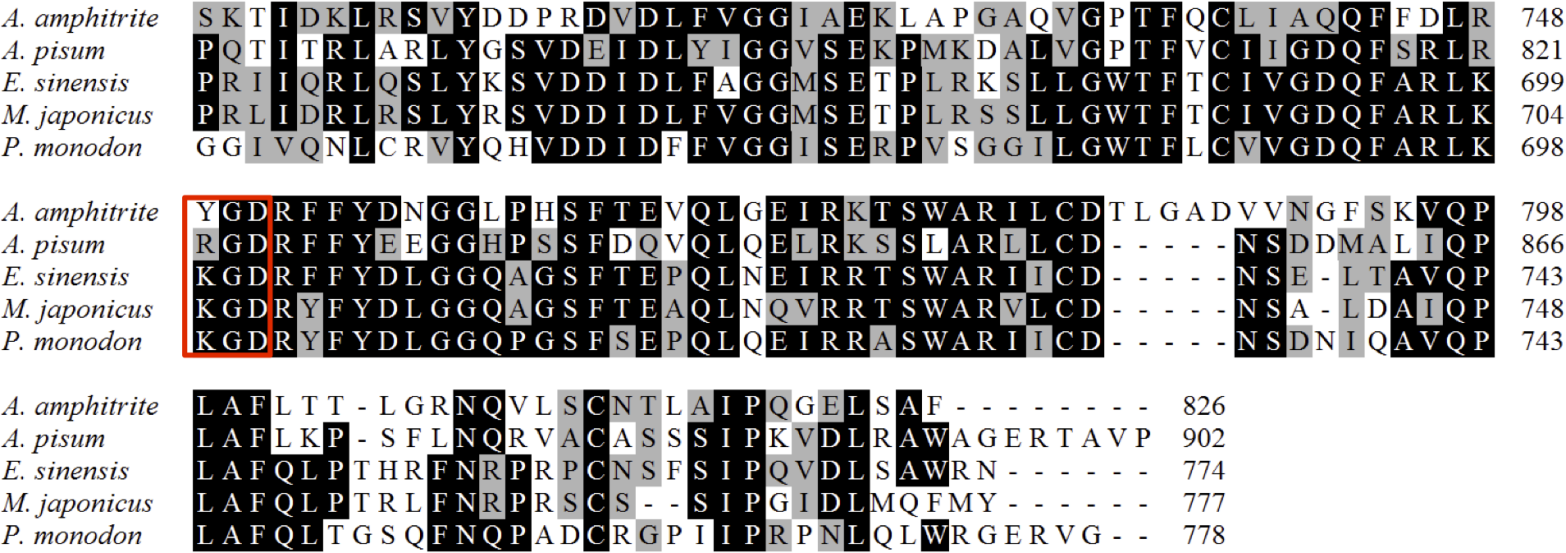
Alignment of the deduced chorion peroxidase/peroxinectin from *Amphibalanus amphitrite*, *Acyrthosiphon pisum* (XP001946672.2), *Eriocheir sinensis* (ADF87945.1), *Macrophthalmus japonicas* (AID47197.1), and *Penaeus monodon* (AAL05973.1). The putative integrin-binding motif is indicated by a red box, and in *A*. *amphitrite*, this motif was mutated to YGD (Tyr-Gly-Asp) rather than the canonical sequence RGD (Arg-Gly-Asp) or KGD (Lys-Gly-Asp).

A protein (coded by CL8390.Contig1_Ba_mix) identified in all three extractions showed 34% identity to the chorion peroxidase from the pea aphid *Acyrthosiphon pisum* (XP001946672.2) and 40% identity to that from the Chinese mitten crab *Eriocheir sinensis* (also named as peroxinectin; ADF87945.1). One putative integrin-binding motif was located at the C terminus of this protein, and this motif was mutated to YGD (Tyr-Gly-Asp) rather than the canonical sequence RGD (Arg-Gly-Asp) or KGD (Lys-Gly-Asp) (Fig 5).

### Immune-related protein

**Fig 6:**
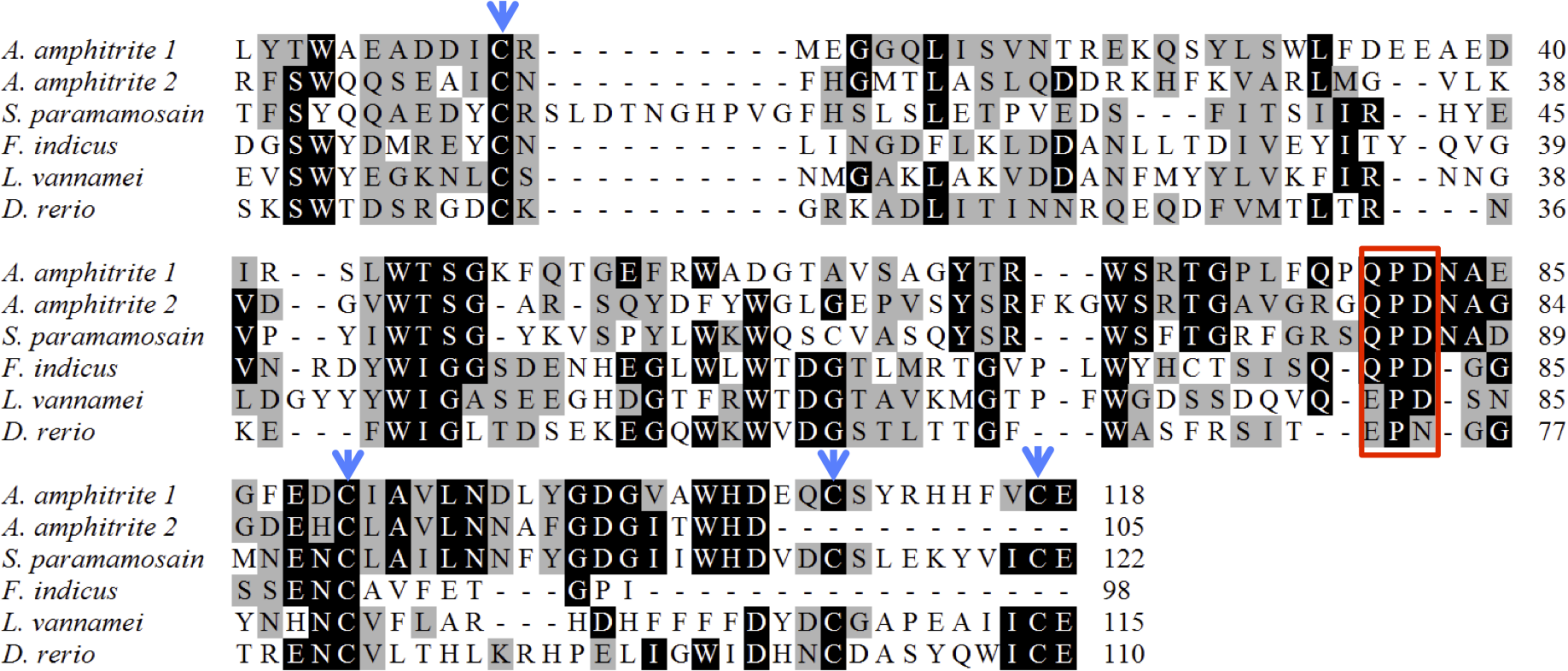
Alignment of C-type lectin-like domains from *Amphibalanus amphitrite* (I and II), *Scylla paramamosain* (ADF27340.1), *Fenneropenaeus indicus* (ADV17348.1), *Litopenaeus vannamei* (AGV68681.1), and *Danio rerio* (XP005172687.1). Similarly to *S*. *paramamosain* and *F*. *indicus*, the typical "EPD" or "EPN" motif in both C-type lectin-like domains (I and II) was mutated into "QPD" in *A*. *amphitrite* (boxed in red). At least two and four cysteines were conserved in the C-type lectin-like domains I and II, respectively. These conserved cysteines are indicated by arrows.

Two proteins containing a C-type lectin-like domain were detected in the shell of *A*. *amphitrite*. Among them, the protein coded by CL14971.Contig1_Ba_mix was present in all three fractions, while the protein coded by Unigene5283_Ba_mix was detected in the 1% SDS fraction. After searching NCBI, both proteins were found to be the most similar to the mannose-binding protein from the mud crab *Scylla paramamosain*, with a similarity of 22.5% and 18.8%, respectively. Alignment with the C-type lectin-like domains from other animals revealed that both C-type lectin-like domains found in this study included a "QPD" motif in the Ca^2+^-binding site and 2 or 4 conserved cysteine residues (Fig 6).

### HE and DAPI staining

**Fig 7:**
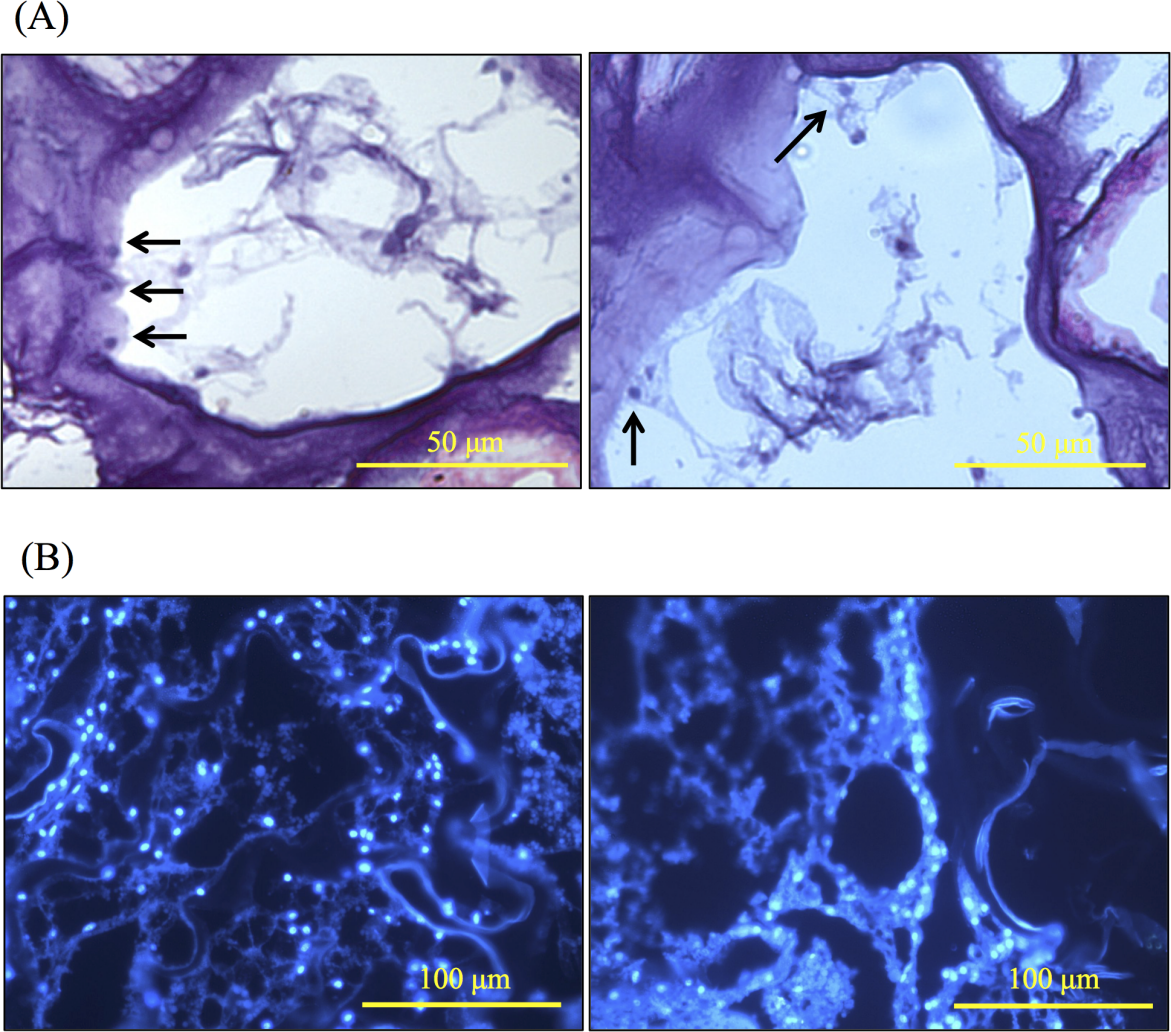
Hematoxylin-eosin (A) and DAPI staining (B) showing cells in histological sections of the *Amphibalanus amphitrite* shell. Arrows identify potential cell structures.

To evaluate the presence of living cells in the barnacle shell, decalcified shells were cut into thin sections and stained with HE or DAPI. At the lower part of the shell paries (just above the basal suture), HE staining revealed cell-shaped structures that were attached to the surfaces of the small inner channels around lacunaes (Fig 7A), and DAPI staining showed cell nucleuses (Fig 7B).

## Discussion

### Chemical component of the barnacle shell

The mineral of barnacle shell is calcite [17] that is the most stable polymorph of calcium carbonate. In this study, the barnacle shell displayed effervescence in 5% acetic acid during decalcification, suggesting the presence of carbonate cement. The XRF analysis revealed high concentration of Ca in the *A*. *amphitrite* shell, which is similar to the shell of mollusks [18, 19], but different from some other crustacean shells, such as shrimp [20], crab, and lobster [21], in which Ca represents only 12–27.8% of the weight of the exoskeleton. A high content of Ca might contribute to the rigidity of the barnacle shell and subsequently protect the soft body.

Gordon et al. [22] showed that Na abundance in the *Balanus* shells varied from 0.16% to 0.50%, and was proportional to environmental salinity; Mn abundance ranged from 0.008% to 0.38%, and was related to the Mn concentration in the local seawater. In this study, Na represented 1.40% of weight percentage, but Mn was not detected. These results are different from Gordon et al. [22], which might be attributed to the related concentrations in the local environment.

In the shells of the barnacle *Balanus balanoides* and *Elminius modestus*, the ratio of Sr:Ca ranges from 7×10^−3^ to 9×10^−3^ [23]. In this study, the ratio of Sr:Ca was equal to 1.17×10^−2^, similarly to those in *B*. *balanoides* and *E*. *modestus*, indicating that ratio of Sr:Ca might be relatively constant among different barnacle species.

### Proteomics analysis of the barnacle shell

Because shell proteins are often insoluble, highly acidic, and interact with minerals to form complexes, it is difficult to extract and purify them directly [24]. In the present study, to minimize the interference of carbonate minerals, barnacle shells were soaked in 5% acetic acid. Subsequently, the residues were extracted using a high concentration of urea, SDS, and DTT. These strong buffers should dissolve as many proteins as possible.

The proteins present in the acetic acid fraction should be relatively hydrophilic, whereas the proteins in the 1% and 10% SDS fractions should be more hydrophobic or cross-linked by disulfide bonds. Six proteins were shared by all three fractions, which suggested that these proteins might be abundant in the shell or present in different forms. For a given protein, some molecules might form complexes with other proteins or substrates, and a strong buffer may be required to dissolve these complexes; in contrast, other molecules might be freely present in epithelium cells and soluble in the acetic acid fraction.

### Settlement-inducing protein complex

SIPC is not only a waterborne pheromone that induces the gregarious settlement of barnacle larvae [7], but also a contact pheromone of importance in species recognition [25], since it can be detected in the footprints deposited by cyprids [6] and the adult shells [8]. SIPC is expressed in the cuticles of both nauplius and cypris larvae as well as the adult, indicating that it is produced by the epidermal cells that secrete the cuticle [8]. The presence of SIPC in the barnacle shell suggested epidermal cells in the barnacle shell.

### Shell matrix proteins

Unusual acidic proteins, such as Aspein and Asprich, have previously been identified in the shell matrix of mollusks [26, 27]. Aspein from the pearl oyster *Pinctada fucata* is rich in Asp (57.6%), Gly (15.5%), and Ser (12.8%) residues (S2 Table), with an isoelectric point (pI) of 1.45. Its sequence is almost completely occupied by poly(Asp) blocks punctuated with Ser-Gly dipeptides [26] (Fig 2). Asprich from the bivalve *Atrina rigida* is composed of 60.3% acidic amino acids (48.6% Asp and 11.7% Glu), with Gly occupying approximately 5.4% of the amino acid sequence (S2 Table). Its average pI is equal to 3.1 [27]. In Asprich, a domain with DEAD repeats displaying Mg^2+^-binding capability was identified [27]. To the best of our knowledge, both Aspein and Asprich have been only reported in bivalves, and not in any crustacean.

In this study, the protein coded by the contig CL6615.Contig1_Ba_mix was identified to be an acidic shell matrix protein, which displayed considerable similarity to the Asprich from *A*. *rigida* and the Aspein from *P*. *fucata*. However, the high similarity of the sequence alignment was not sufficient to confirm that this protein was Asprich or Aspein, because all of them were phylogenetically uninformative. All of these proteins contained extensive low-complexity regions (rich in Asp, Glu, or Gly), and phylogenetic alignment of these proteins would lead to false-positive results [28]. Based on the amino acid composition and sequence alignment, this acidic shell matrix protein was much more similar to Asprich than to Aspein. Nevertheless, it lacked either the typical "polyD punctured with SG" structure in Aspein (Fig 2) or the conserved DEAD motif in Asprich (Fig 2). Thus, we considered it to be a novel acidic shell matrix protein. Asp is associated with calcium-binding and possibly magnesium-binding activity through interactions with the carboxylate groups of Asp [27]. Poly(Glu) domains are responsible for protein aggregation [29]. High contents of Asp and poly(Glu) in the acidic shell matrix protein indicated that this protein might participate in the formation of the shell structure.

Prisilkin-39 was first detected in the shell of *P*. *fucata* [30], and then its homolog was found in fish (*Cynoglossus semilaevis*, XP008334378) and insect (*Nasonia vitripennis*, XP003424571). This protein is a highly repetitive protein with a high composition of Gly (29.72%), Tyr (23.26%), and Ser (18.60%) residues [30]. In the shell of *P*. *fucata*, Prisilkin-39 binds tightly with chitin, an insoluble polysaccharide that forms the highly structured framework of the shell and is involved in chitinous framework building. It also participates in the regulation of crystal growth during prismatic layer mineralization [30]. In the present study, the protein coded by the contig CL4062.Contig1_Ba_mix was identified to be Prisilkin-39 with an identity of 44%, and has a basic isoelectric point, indicating that this protein might bind to chitin and provide a complementary function to the acidic shell matrix protein mentioned above, forming the basis of the barnacle shell.

### Serine protease and proteinase inhibitor protein

Barnacle shell is considered to consist of only acellular biominerals. It is puzzling to detect proteases and proteinase inhibitor proteins in the shell. However, this is not an isolated observation [31]. These proteins/domains have been identified in the shell matrix of the abalone *H*. *refescens*, *H*. *asinia*, and *H*. *laevigata*, the pearl oyster *P*. *margaritifera* and *P*. *fucata*, the sea urchin *Strongylocentrotus purpuratus* as well as chicken eggs (*Gallus gallus domesticus*) (summarized in [31]).

Similar to trypsin and prostate-specific antigen (PSA), serine proteases are likely synthesized as a preproenzyme containing an N-terminal signal peptide followed by a short activation peptide and an enzymatic domain at the C-terminal end [14]. In barnacle, shell formation is in a dynamically steady state balanced by shell construction and molting. The trypsin-like serine protease might, similar to that in human bone [32], function to digest extracellular matrix proteins for shell resorption. As a cofactor, serine protease cascades also participate in the proteolytic activation of prophenoloxidase (proPO), which is a reaction implicated in melanotic encapsulation, wound healing, and protein cross-linking [33]. These serine proteases might also play a role in the protection in the barnacle shell. Moreover, serine proteases might also take a role in the hardening process of barnacle shell, as these proteases have already been shown to participate in cement polymerization [34].

Purified proteinase inhibitor proteins from the nacre of the oyster *P*. *margaritifera* revealed specific inhibitory activity to serine proteases [35]. Thus, these inhibitor proteins might play a role in either protection of proteins involved in shell formation or in defense against parasites, or both [35]. In this study, the chorionic proteinase inhibitor might have similar functions, either regulating the shell formation or functioning in immunity. Moreover, the chorionic protease inhibitor found in the barnacle shell had 5 WAP domains, suggesting that chorionic protease inhibitor might have negative effects on crystal formation, as 3-repeated WAP domains in the perlwapin from the sea snail *H*. *laevigata* shell inhibited the growth of certain crystallographic planes during biomineralization [36].

### Carbonic anhydrase

Carbonic anhydrase, that catalyzes the hydration of CO_2_ to produce HCO_3_^-^ and H^+^, is essential for the calcification process. It has been identified in several calcifying epithelia as well as some extracellular skeletal matrices (such as crustacean calcium storage concretions and mollusk shells) [37]. In the barnacle shell, three homologs of carbonic anhydrase were detected, which might be associated with the production of bicarbonate ions to precipitate calcium carbonate; it might also be possible that carbonic anhydrase produces protons and then induces the dissolution of calcium carbonate [37]. For carbonic anhydrase I and III, two of the three histidines that function as zinc ligands were mutated, revealing that carbonic anhydrases I and III might function in other ways or possess different activities.

### Chorion peroxidase

Chorion peroxidase participates in the hardening process (cuticular tanning) of the chorion in the mosquito *Aedes aegypti* by catalyzing chorion protein crosslinking through dityrosine formation [38]. A contig (CL8390.Contig1_Ba_mix) in all three fractions was identified as chorion peroxidase in the barnacle shell, which has a mutated motif of YGD rather than RGD or KGD. This mutated YGD motif also displays the ability to bind integrin [39]. In the barnacle shell, the outer surface is covered by a cuticle, and the organic components of the shelly layers represent part of the endocuticle [40]. It is possible that chorion peroxidase may function in the cuticle hardening process in the shell.

### C-type lectins

C-type lectins or C-type lectin-like proteins/domains are widely present in biominerals [41]. These conserved domains are mainly responsible for calcium-dependent carbohydrate binding. In addition, they bind selectively to various ligands, including lipids, proteins and inorganic compounds (such as CaCO_3_ and ice) [42]. They are also involved in the recognition and clearance of microbial agents in both vertebrate and invertebrate immunity [43]. In the barnacle *Megabalanus rosa*, C-type lectins participate in the mineralization and defense in shells [44]. *In vitro* studies revealed that C-type lectins inhibited the crystal nucleation of aragonite and regulated the morphology and growth orientation of calcite through binding to the surface of the crystal to stop its growth [45]. In this study, C-type lectin-like domains in the shell of *A*. *amphitrite* might also has the activity in the regulation of crystal formation.

Moreover, the EPN and QPD motif is important for mannose and galactose binding, respectively [46]. In the present study, two proteins containing a C-type lectin-like domain were detected in the shell of *A*. *amphitrite*. The presence of a conserved QPD motif suggested that these two domains in *A*. *amphitrite* might be important for the detection of galactose in bacteria and participate in the immune defense mechanisms.

### HE and DAPI staining of barnacle shell

As previously reported, epithelial cells were present in the exoskeletons of crustaceans and the shells of mollusks. Further studies confirmed their participation in the shell formation processes [47, 48]. In barnacle shells, the regions of growth comprise the basal suture (the joint between the lower outer edge of the basis and lower parts of the paries), the downward protruding region of the sheath, the margins of the opercular plates, the radii and the alae [40]. Later histological studies observed elongated cuticle-secreting cells in the basal suture, and variously modified hypodermal cells underlying the basis, the opercular membrane, and the shell plates [40]. Recently, Gohad et al. [49] found chloride epithelia at the basis and parietal plates of adult barnacles using silver staining microscopic techniques. In the present study, HE and DAPI staining revealed cell-shaped structures attaching to the surfaces of the inner channels around lacunae, which is consistent with the findings by Gohad et al. [49]. Proteomics analysis identified 9 cytoskeletal proteins and several metabolism-related intracellular proteins such as ATP synthase and fructose 1, 6-bisphosphate aldolase, which are considered to only function intracellularly. These results further confirmed the existence of epithelia in the shell parietes.

## Conclusions

Using XRF analysis, we found that Na, Mg, Ca, and Sr represented 1.4%, 1.0%, 93.8%, and 1.1% of weight percentage in the *A*. *amphitrite* shell. Proteomics results detected 52 proteins in the barnacle shell. Based on KOG database, 40 proteins were categorized into 11 functional groups. Besides some known shell proteins (SIPC, acidic shell matrix proteins, and carbonic anhydrase), we also detected a basic shell matrix protein (Prisilkin-39), C-type lectin-like domains, chorion peroxidase, serine proteases and a proteinase inhibitor protein in the barnacle shell. The HE and DAPI staining revealed living cells in the shells, which might secrete the shell proteins.

## Materials and Methods

### Ethics Statement

No specific permit is required for the present study in Hong Kong.

The place for barnacle collection does not belong to any national parks, protected areas or private lands. Barnacle collection there does not involve any endangered or protected species or contravene any environmental protection law.

### Barnacle collection and shell preparation

Adults of *Amphibalanus amphitrite* were collected from Sai Kung Pier, Hong Kong (22°22'53''N, 114°16'32''E). After the removal of soft tissues and basal plates, the outer and inner surfaces of the shell were carefully brushed with 0.22 μm-filtered seawater (FSW). Next, the shells were immersed in sodium hypochlorite (active chlorine≥6.25%) at 4°C overnight to eliminate trace contamination of soft tissue. After flushing with Mill-Q water and air-drying, the shells were ready for further analysis.

### X-ray fluorescence analysis

Bleached shells were ground into powder, heated in an oven at 110°C for 10 hours to remove moisture, and finally examined using an X-ray fluorescence (XRF) spectrometer (JEOL JSX-3201Z) to analyze the chemical components of the barnacle shell.

### Decalcification and protein extraction

Shells were ground into fine particles in liquid nitrogen and then decalcified in 5% acetic acid at 4°C overnight. After centrifugation at 10,000 g for 20 min, the pellet was further washed twice in 5% acetic acid. All of the supernatants were pooled together as acetic acid fraction.

The pellets containing acid-insoluble proteins were first extracted in 1% SDS extraction buffer (8 M urea, pH 7.4, 0.5 M DTT, 1% SDS and commercial protease inhibitor, Roche, German) and then further extracted in 10% SDS extraction buffer (8 M urea, pH 7.4, 0.5 M DTT, 10% SDS and commercial protease inhibitor, Roche, German). Following each extraction, the pellets were washed twice in the same buffer used.

### Polyacrylamide gel electrophoresis and in-gel digestion

The protein concentrations were determined using a RC-DC protein assay kit (Bio-Rad, CA, USA). All of the samples were loaded on 12% polyacrylamide gel (PAGE). For each fraction, 80 μg of total protein was loaded in the gel. After coomassie blue staining, the lane of each fraction was cut into 10 fragments for total protein profiling. The in-gel digestion procedure has been described by Zhang et al. [50].

### Mass spectrometric analysis and Mascot searching

After a clean-up procedure using ZipTip C18 peptide tips (Millipore, MA),the peptides were analyzed using an electrospray ionization (ESI)-quadrupole time-of-flight (Q-TOF) mass spectrometer (QSTAR XL, Applied Biosystems/Sciex, ON, Canada). Another biological replicate of sample was prepared using sample procedure and finally analyzed using a LTQ Velos Dual-Pressure Ion Trap Mass Spectrometer (coupled with LC and ETD source, Thermo Scientific). The protocols for Q-TOF and LTQ-MS analysis have been described by Han et al. [51] and Liu et al. [52], respectively. Each sample was injected twice and the results were combined together.

The raw data files generated by ESI-QqTOF were converted into.*pkl* format using ProteinLynx (v2.2.5, waters). MASCOT daemon (v2.2) was applied to search the peptide information against our in-house transcriptome database of *A*. *amphitrite* [53]. Decoy sequences generated by Trans-Proteomic Pipeline (TPP) were added in the database [54] and the protein identification false discovery rate (FDR) cutoff was set at 0.01.

### Bioinformatics analysis

The proteins were categorized into different functional groups via a tBLASTx search against the KOG database [55]. Potential motifs in each gene were scanned using the SMART website (http://smart.embl-heidelberg.de). The physical and chemical parameters of the proteins were estimated using the ProtParam tool available on the ExPASy Bioinformatics Resource Portal (http://web.expasy.org). The identity and similarity between proteins were calculated using SIAS (http://imed.med.ucm.es/Tools/sias.html). Signal peptides were predicted online using the SignalP 4.1 Server (http://www.cbs.dtu.dk/services/SignalP/).

### Histological sections, hematoxylin-eosin and DAPI staining

The bleached shells were fixed in 4% PFA/PBS solution at 4°C overnight and then decalcified in 0.5 M EDTA (pH 8.0) at 4°C until they became soft. After three washes with PBS, the samples were dehydrated in gradient concentrations of ethanol, infiltrated in xylene and embedded in wax. The samples were cut into 4-μm-thick sections and mounted onto glass slides. After dewaxing using xylene, the sections were rehydrated in gradient concentrations of ethanol followed by PBS.

The HE staining was performed using a HE staining kit (Jiancheng Biotech., Nanjing, China). Subsequently, the sections were sealed using DPX mountant for histology (Sigma) and observed under a light microscopy.

Rehydrated sections were stained with 300 nM DAPI for 30 min, and then washed with PBS for 4×15 min. Next, the sections were sealed and observed under a fluorescent microscopy.

## Supporting Information

S1 Appendix DNA sequences of the contigs mentioned in this study. (TXT)

S1 Fig X-ray spectrum of the *Amphibalanus amphitrite* shell. High concentrations of Ca, Cl, S, Sr, Mg and Na elements were detected in the barnacle shell.
(TIFF)

S2 Fig A Venn diagram representing the overlap among the three fractions of proteins extracted from the *Amphibalanus amphitrite* shell. In total, 52 proteins were identified in all three fractions, and 20, 3, and 12 proteins were uniquely detected in the acetic acid, 1% SDS and 10% SDS fractions, respectively.
(TIFF)

S1 Table Proteins identified in the *Amphibalanus amphitrite* shell. The Genbank access number and protein description identify protein matches from the NCBI database. The NCBI score and E-value are obtained from the tBLASTx searching. The protein score is derived from the combined scores of all observed mass spectra that can be matched to amino acid sequences within that protein. Protein matches gives the number of mass spectra assigned to this protein. ✓ means present in that fraction.
(PDF)

S2 Table Amino acid composition of the Asp-rich shell matrix protein from *Amphibalanus amphitrite*, Asprich from *Atrina rigida* and Aspein from *Pinctada fucata*. (PDF)
