# Supplementary figures and images for: Chemical Component and Proteomic Study of the Amphibalanus (= Balanus) amphitrite Shell
Source: PLoS One. 2015 Jul 29;10(7):e0133866. doi: 10.1371/journal.pone.0133866 (PMC4519255; doi:10.1371/journal.pone.0133866)

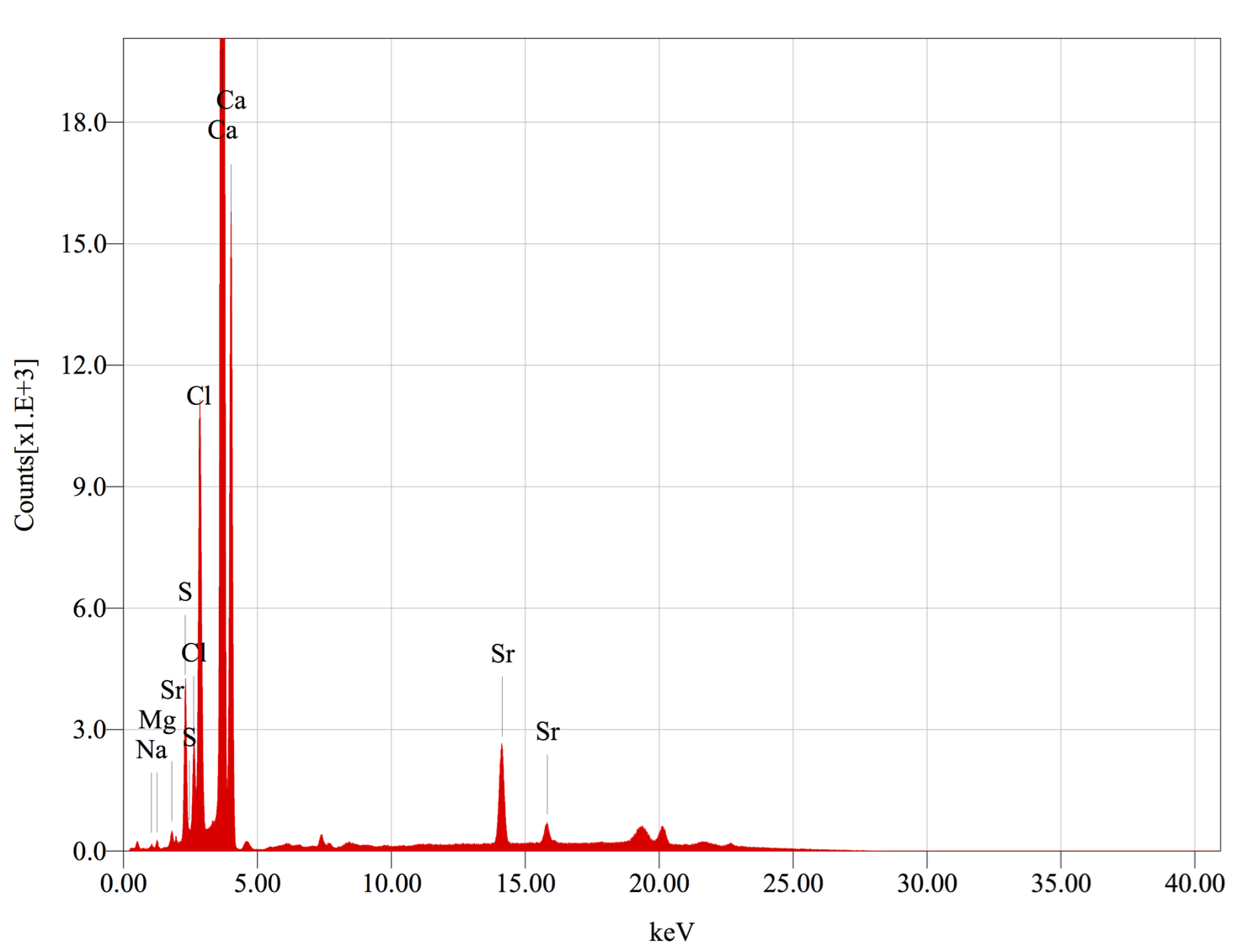

Supplement: S1 Fig — High concentrations of Ca, Cl, S, Sr, Mg and Na elements were detected in the barnacle shell. (TIFF) [file pone.0133866.s002.tiff]

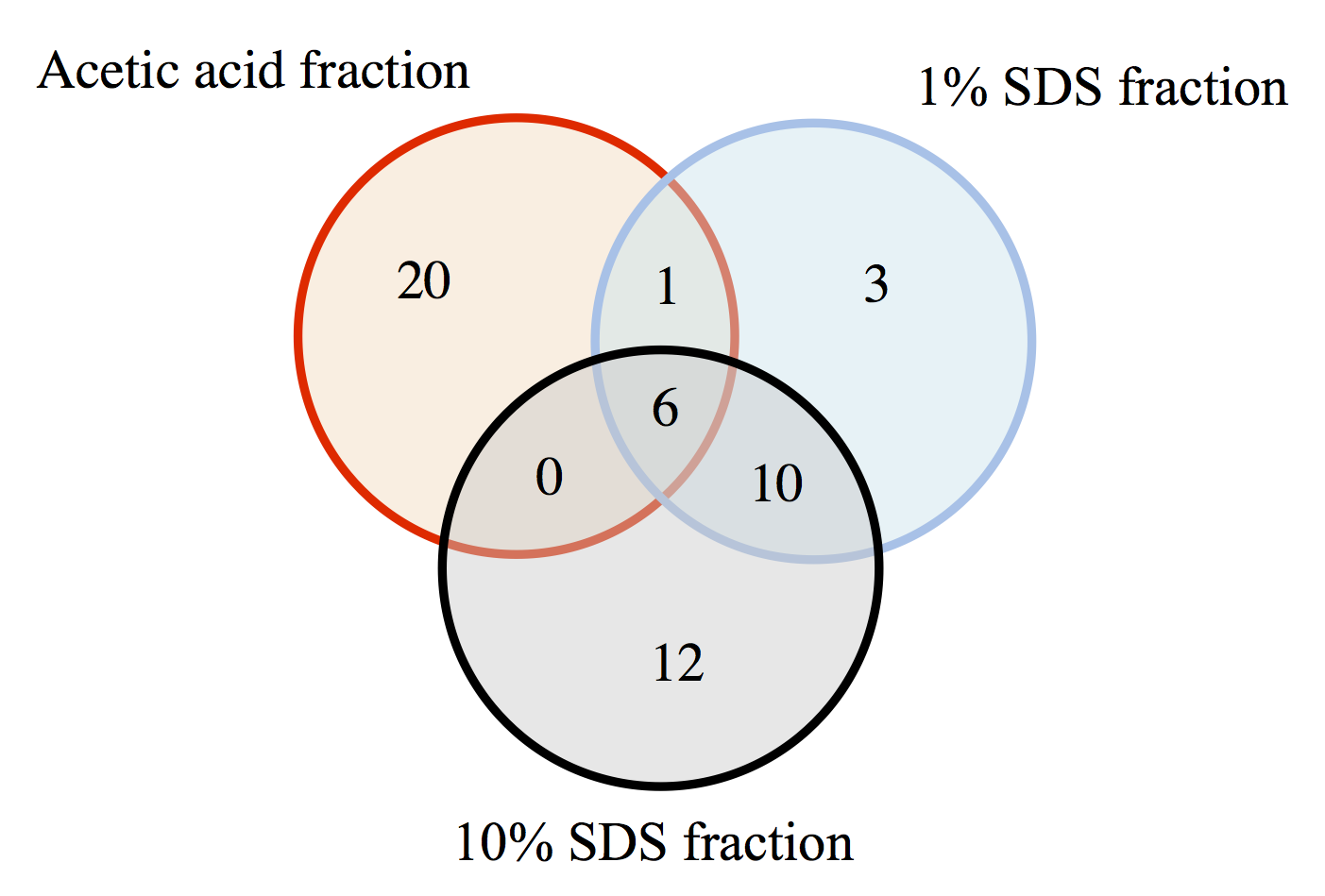

Supplement: S2 Fig — In total, 52 proteins were identified in all three fractions, and 20, 3, and 12 proteins were uniquely detected in the acetic acid, 1% SDS and 10% SDS fractions, respectively. (TIFF) [file pone.0133866.s003.tiff]
