# Supplementary material for: Chemical Component and Proteomic Study of the Amphibalanus (= Balanus) amphitrite Shell
Source: PLoS One. 2015 Jul 29;10(7):e0133866. doi: 10.1371/journal.pone.0133866 (PMC4519255; doi:10.1371/journal.pone.0133866)
Supplement: S1 Table — The Genbank access number and protein description identify protein matches from the NCBI database. The NCBI score and E-value are obtained from the tBLASTx searching. The protein score is derived from the combined scores of all observed mass spectra that can be matched to amino acid sequences within that protein. Protein matches gives the number of mass spectra assigned to this protein. ✓ means present in that fraction. (PDF) [file pone.0133866.s004.pdf]

Table S1. Proteins identified in the Amphibalanus amphitrite shell. The Genbank access number and protein description identify protein matches from the NCBI database. The NCBI score and E-value are obtained from the tBLASTx searching. The protein score is derived from the combined scores of all observed mass spectra that can be matched to amino acid sequences within that protein. Protein matches gives the number of mass spectra assigned to this protein. ✓ means present in that fraction.

Contig number	Function discription	NCBI number	NCBI score	NCBI E-value	Protein score	Peptide matches	Fraction		
							Acetic acid	1% SDS	10% SDS
**Chromatin structure and dynamics**									
Unigene28047_Ba_mix	histone-lysine N-methyltransferase MLL2-like	XP002935167.1	90.1	6.E-17	156	11	✓		
**Energy production and conversion**									
CL215.Contig2_Ba_mix	F0F1-type ATP synthase, beta subunit	XP001996493.1	847	0.E+00	48	4			✓
CL15717.Contig1_Ba_mix	Iron binding protein involved in Fe-S cluster formation	XP002739643.1	175	1.E-42	35	5	✓		
**Amino acid transport and metabolism**									
CL1641.Contig1_Ba_mix	serine proteinase-like protein	ABD62888.1	287	2.E-75	205	10		✓	✓
CL200.Contig3_Ba_mix	serine proteinase-like protein	ABD62888.1	348	8.E-94	120	13		✓	✓
CL389.Contig2_Ba_mix	pacifastin light chain precursor	AAC64661.1	80.1	2.E-13	38	8	✓		
**Lipid transport and metabolism**									
CL775.Contig1_Ba_mix	vitellogenin 2	ACJ12892.1	327	4.E-87	2241	397		✓	✓
CL775.Contig2_Ba_mix	vitellogenin	BAJ33507.1	233	4.E-59	991	169		✓	✓
CL565.Contig1_Ba_mix	vitellogenin 2	ACJ12892.1	246	5.E-82	824	77		✓	✓
**Posttranslational modification, protein turnover, chaperones**									
CL68.Contig2_Ba_mix	Settlement inducing protein complex	AAR33079.1	2979	0.E+00	425	68		✓	✓
CL17587.Contig1_Ba_mix	Alpha-macroglobulin	EFN79621.1	573	0.E+00	164	26			✓

Contig number	Function discription	NCBI number	NCBI score	NCBI E-value	Protein score	Peptide matches	Fraction		
							Acetic acid	1% SDS	10% SDS
Unigene28512_Ba_mix	Basement membrane-specific heparan sulfate proteoglycan (HSPG) core protein	EFN62243.1	222	1.E-56	249	17			✓
CL1258.Contig1_Ba_mix	AGAP003656-PA	XP_972068.2	382	1.E-172	122	3			✓
CL7049.Contig1_Ba_mix	Basement membrane-specific heparan sulfate proteoglycan (HSPG) core protein	EFN62243.1	211	8.E-53	112	9			✓
CL7056.Contig1_Ba_mix	papilin-like	XP001601735.2	189	1.E-46	112	6	✓		
CL6376.Contig1_Ba_mix	GA22193	XP002136422.1	126	2.E-27	119	6	✓		
Unigene7974_Ba_mix	similar to AGAP003656-PA	XP972068.2	273	9.E-72	65	8			✓
CL5577.Contig1_Ba_mix	chorionic proteinase inhibitor	BAC00855.1	124	5.E-27	35	11	✓		
**General function prediction only**									
CL8390.Contig1_Ba_mix	Chorion peroxidase-like	XP001946672.2	440	1.E-121	1375	377	✓	✓	✓
Unigene8560_Ba_mix	Carboxylesterase and related proteins	NP001136104.1	75.9	2.E-12	294	29			✓
Unigene27659_Ba_mix	carbonic anhydrase 2	NP001182637.1	113	2.E-23	102	16		✓	✓
CL15286.Contig1_Ba_mix	alpha-carbonic anhydrase	EFX81683.1	123	2.E-26	127	4	✓	✓	✓
CL10121.Contig1_Ba_mix	alpha-carbonic anhydrase	EFX88013.1	149	5.E-34	69	9		✓	✓
**Signal transduction mechanisms**									
CL14971.Contig1_Ba_mix	mannose-binding protein	AAX55747.1	111	1.E-22	235	59	✓	✓	✓
CL333.Contig5_Ba_mix	AGAP003027-PA	EAA07856.5	396	1.E-108	150	10	✓		
Unigene5283_Ba_mix	mannose-binding protein	ADF27340.1	78.6	1.E-13	93	3		✓	
Unigene22882_Ba_mix	STE20-like serine/threonine-protein kinase-like isoform 3	XP003401043.1	582	1.E-164	47	22	✓		
Unigene22883_Ba_mix	STE20-like serine/threonine-protein kinase-like isoform 3	XP003401043.1	376	1.E-102	49	20	✓		
**Defense mechanisms**									

Contig number	Function discription	NCBI number	NCBI score	NCBI E-value	Protein score	Peptide matches	Fraction		
							Acetic acid	1% SDS	10% SDS
Unigene23219_Ba_mix	sushi, nidogen and EGF-like domain-containing protein 1	XP002750027.1	58.2	2.E-07	192	22	✓		
**Extracellular structures**									
CL9487.Contig1_Ba_mix	similar to Fraser syndrome 1 isoform 1	XP535623.2	73.6	1.E-11	160	4	✓		
CL4062.Contig1_Ba_mix	Prisilkin-39	C0J7L8	72.4	8.E-12	151	6	✓	✓	✓
CL6615.Contig1_Ba_mix	acetic shell matrix protein	BAD00044.1	333	1.E-89	70	3	✓		
**Cytoskeleton**									
CL3592.Contig1_Ba_mix	Actin	CAB99474.1	762	0.E+00	502	101		✓	✓
Unigene32973_Ba_mix	Actin	XP623487.2	424	1.E-117	273	64			✓
Unigene11591_Ba_mix	Myosin class II heavy chain	XP003395921.1	1135	0	47	14			✓
CL158.Contig4_Ba_mix	beta-tubulin	XP002110118.1	900	0	32	7			✓
CL1283.Contig1_Ba_mix	Titin	EFN80688.1	892	0	63	29	✓		
CL352.Contig1_Ba_mix	Talin-1	EGI67012.1	3195	0	54	16	✓		
CL6344.Contig2_Ba_mix	Myosin class II heavy chain	XP003397476.1	3014	0	55	25	✓		
**Cell cycle control, cell division, chromosome partitioning**									
CL1818.Contig1_Ba_mix	Mki67 protein	AAI21193.1	191	1.E-46	44	34			✓
**Function unknown**									
Unigene27606_Ba_mix	Basic proline-rich protein	Q95JC9	216	3.E-54	612	119	✓	✓	✓
Unigene27724_Ba_mix	Hypothetical protein	--	--	--	190	11	✓	✓	✓
Unigene28390_Ba_mix	Hypothetical protein	--	--	--	119	4		✓	
CL2023.Contig1_Ba_mix	GE11827	XP002092149.1	108	4.E-22	149	6	✓		
CL5140.Contig1_Ba_mix	AGAP002085-PA	XP320958.3	86.3	2.E-15	119	11	✓		
Unigene5172_Ba_mix	Hypothetical protein CBG20711	XP002631543.1	76.6	1.E-12	87	3	✓		
Unigene30333_Ba_mix	Hypothetical protein	--	--	--	61	6	✓	✓	
CL3876.Contig2_Ba_mix	CG2839	NP608540.1	101	5.E-20	61	5	✓		
Unigene10933_Ba_mix	Hypothetical protein	--	--	--	47	8	✓		

Contig number	Function discription	NCBI number	NCBI score	NCBI E-value	Protein score	Peptide matches	Fraction		
							Acetic acid	1% SDS	10% SDS
Unigene20930_Ba_mix	Hypothetical protein	--	--	--	45	3	✓		
Unigene27308_Ba_mix	Hypothetical protein	--	--	--	41	8			✓
CL6664.Contig1_Ba_mix	CBG02561	XP002630848.1	107	2.E-21	56	12		✓	✓
