# Supplementary material for: Chemical Component and Proteomic Study of the Amphibalanus (= Balanus) amphitrite Shell
Source: PLoS One. 2015 Jul 29;10(7):e0133866. doi: 10.1371/journal.pone.0133866 (PMC4519255; doi:10.1371/journal.pone.0133866)
Supplement: S2 Table — (PDF) [file pone.0133866.s005.pdf]

Table S2. Amino acid composition of the Asp-rich shell matrix protein from *Amphibalanus amphitrite*, Asprich from *Atrina rigida* and Aspein from *Pinctada fucata*.

Amino acid	Asp-rich shell matrix prtein (*A. amphitrite*)	Asprich (*A. rigida*)	Aspein (*P. fucata*)
Ala	5.8	13.6	6.3
Arg	0.3	0.4	0.0
Asn	3.9	2.7	0.5
Asp	41.2	48.6	57.6
Cys	0.0	0.0	0.2
Gln	1.8	0.4	0.5
Glu	21.8	11.7	1.5
Gly	14.2	5.4	15.5
His	0.0	0.4	0.0
Ile	0.5	1.2	0.5
Leu	0.3	2.3	1.0
Lys	1.6	1.2	0.2
Met	0.0	0.4	0.5
Phe	0.3	0.4	0.2
Pro	0.3	1.2	0.5
Ser	4.5	5.8	12.8
Thr	0.3	0.8	0.7
Trp	0.0	0.0	0.0
Tyr	1.8	0.0	0.0
Val	1.6	3.5	1.5
Pyl	0.0	0.0	0.0
Sec	0.0	0.0	0.0
